# Supplementary material for: Social isolation modulates appetite and avoidance behavior via a common oxytocinergic circuit in larval zebrafish
Source: Nat Commun. 2022 May 11;13:2573. doi: 10.1038/s41467-022-29765-9 (PMC9095721; doi:10.1038/s41467-022-29765-9)
Supplement: Supplementary file 1 — Supplementary Information [file 41467_2022_29765_MOESM1_ESM.pdf]

SUPPLEMENTARY FIGURE 1

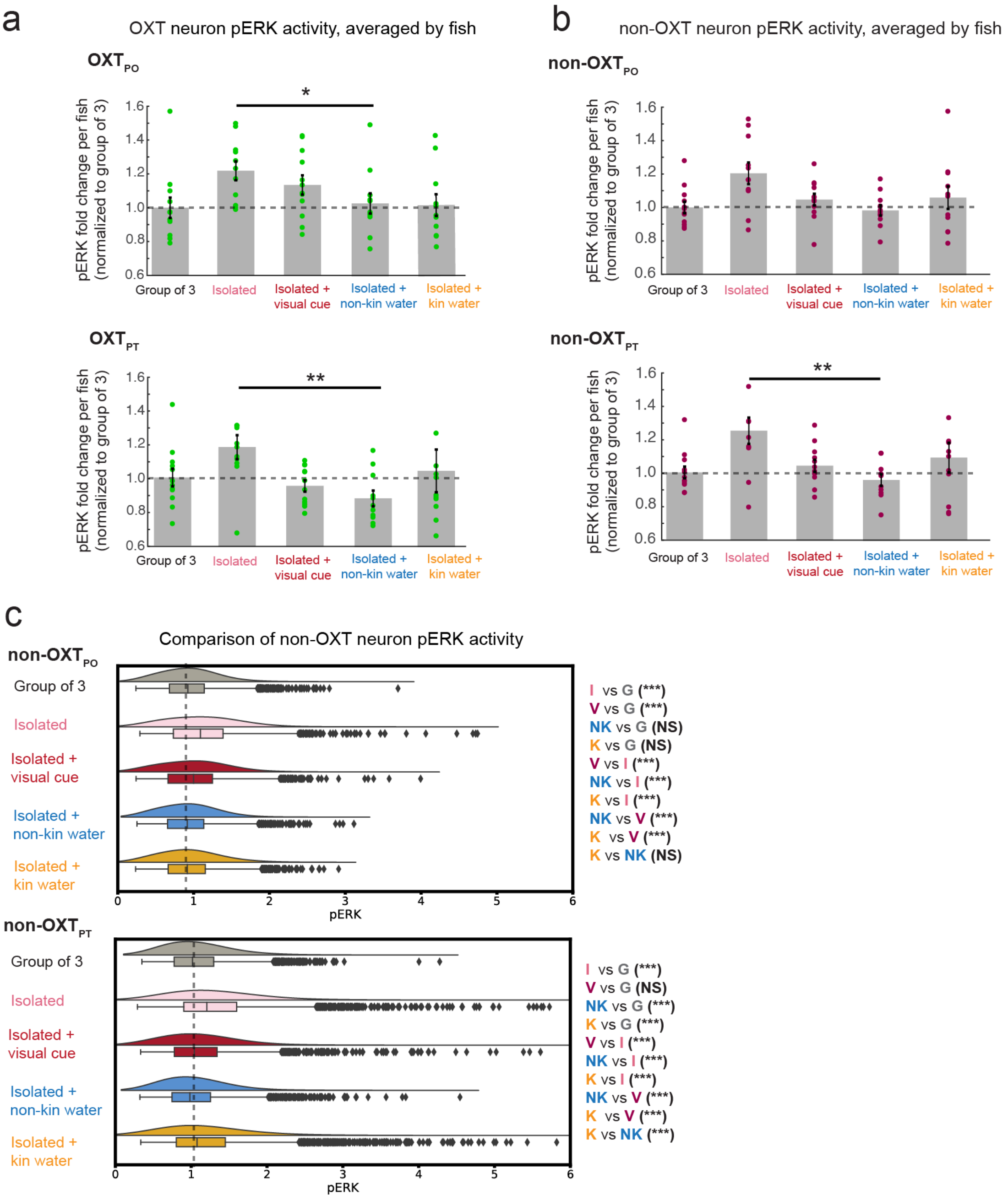

Supplementary Figure 1: Modulation of nearby non-OXT neurons by social context and comparison with OXT neuron activity

Effect of social isolation and social sensory cues on mean OXT_{PO} and OXT_{PT} neuron activity per fish. Data are presented as mean values ± SEM; n = 12 (group) / 11 (isolated) / 12 (visual) / 11 (non-kin) / 11 (kin) fish.

OXT_{PO} neurons: Adjusted p= 0.12 (group vs isolated) / 0.75 (group vs visual) / 0.88 (group vs non-kin water) / 1 (group vs kin water) / 0.75 (isolated vs visual) / 0.01* (isolated vs non-kin

water) / 0.12 (isolated vs kin water) / 0.21 (visual vs non-kin water) / 0.74 (visual vs kin water) / 0.91 (kin vs non-kin water), Kruskal-Wallis Test with Tukey-Kramer Correction for Multiple Comparisons.

OXT_{PT} neurons: p= 0.25 (group vs isolated) / 0.99 (group vs visual) / 0.26 (group vs non-kin water) / 1.0 (group vs kin water) / 0.09 (isolated vs visual) / 8.8×10^{-4}*** (isolated vs non-kin water) / 0.15 (isolated vs kin water) / 0.58 (visual vs non-kin water) / 1.0 (visual vs kin water) / 0.44 (kin vs non-kin water). Kruskal-Wallis Test with Tukey-Kramer Correction for Multiple Comparisons.

a) Effect of social isolation and social sensory cues on mean non-OXT_{PO} and non-OXT_{PT} neuron activity per fish. Sample sizes are the same as in 1a. Data are presented as mean values ± SEM.

non-OXT_{PO} neurons: Adjusted p = 0.13 (group vs isolated) / 0.78 (group vs visual) / 1 (group vs non-kin water) / 1 (group vs kin water) / 0.73 (isolated vs visual) / 0.13 (isolated vs non-kin) / 0.26 (isolated vs kin water) / 0.78 (visual vs non-kin water) / 0.93 (visual vs kin water) / 0.99 (kin vs. non-kin water). Two-sided Kruskal-Wallis Test with Tukey-Kramer correction for multiple comparisons.

non-OXT_{PT} neurons: Adjusted p = 0.051 (group vs isolated) / 0.90 (group vs visual) / 0.99 (group vs non-kin water) / 0.83 (group vs kin water) / 0.35 (isolated vs visual) / 0.014* (isolated vs non-kin) / 0.48 (isolated vs kin water) / 0.65 (visual vs non-kin water) / 1.0 (visual vs kin water) / 0.55 (kin vs. non-kin water). Two-sided Kruskal-Wallis Test with Tukey-Kramer correction for multiple comparisons.

b) Probability distributions and box plots of normalized pERK fluorescence across all neurons per group. Boxplot shows the median (center), interquartile range (IQR, box), 1.5 IQRs of the lower and upper quartile (whiskers), and outliers beyond this range (diamonds); Half-violin plot shows kernel density estimate. Fish were either kept in groups of 3 (gray, n = 6700 non-OXT_{PO} / 5678 non-OXT_{PT} neurons from 12 fish), isolated (pink, n = 6594 non-OXT_{PO} / 4962 non-OXT_{PT} neurons from 11 fish), isolated but exposed to visual cues of conspecifics (red, n = 5157 non-OXT_{PO} / 4081 non-OXT_{PT} neurons from 12 fish) or isolated but exposed to non-kin-conditioned water (blue, n =5099 OXT_{PO} / 4235 OXT_{PT} neurons from 11 fish) or kin-conditioned water (orange, n= 4574 non-OXT_{PO} / 4621 non-OXT_{PT} neurons from 11 fish).

OXT_{PO} neurons: Adjusted p = 0*** (group vs isolated) / 1.8×10^{-15}*** (group vs visual) / 0.44 (group vs non-kin water) / 1 (group vs kin water) / 0*** (isolated vs visual) / 0*** (isolated vs non-kin water) / 0*** (isolated vs kin water) / 3.4×10^{-20}*** (visual vs non-kin water) / $5.7 \times 10^{-}$

^{12}*** (visual vs kin water) / 0.37 (kin vs non-kin water). Two-sided Kruskal-Wallis Test with Tukey-Kramer correction for multiple comparisons.

OXT_{PT} neurons: Adjusted p = 0*** (group vs isolated) / 0.12 (group vs visual) / $6.0x10^{-4}$*** (group vs non-kin water) / $3.8x10^{-19}$*** (group vs kin water) / 0*** (isolated vs visual) / 0*** (isolated vs non-kin water) / 0*** (isolated vs kin water) / $3.1x10^{-8}$*** (visual vs non-kin water) / $1.4x10^{-8}$*** (visual vs kin water) / 0*** (kin vs non-kin water). Two-sided Kruskal-Wallis Test with Tukey-Kramer correction for multiple comparisons.

Source data are provided as a Source Data file.

SUPPLEMENTARY FIGURE 2

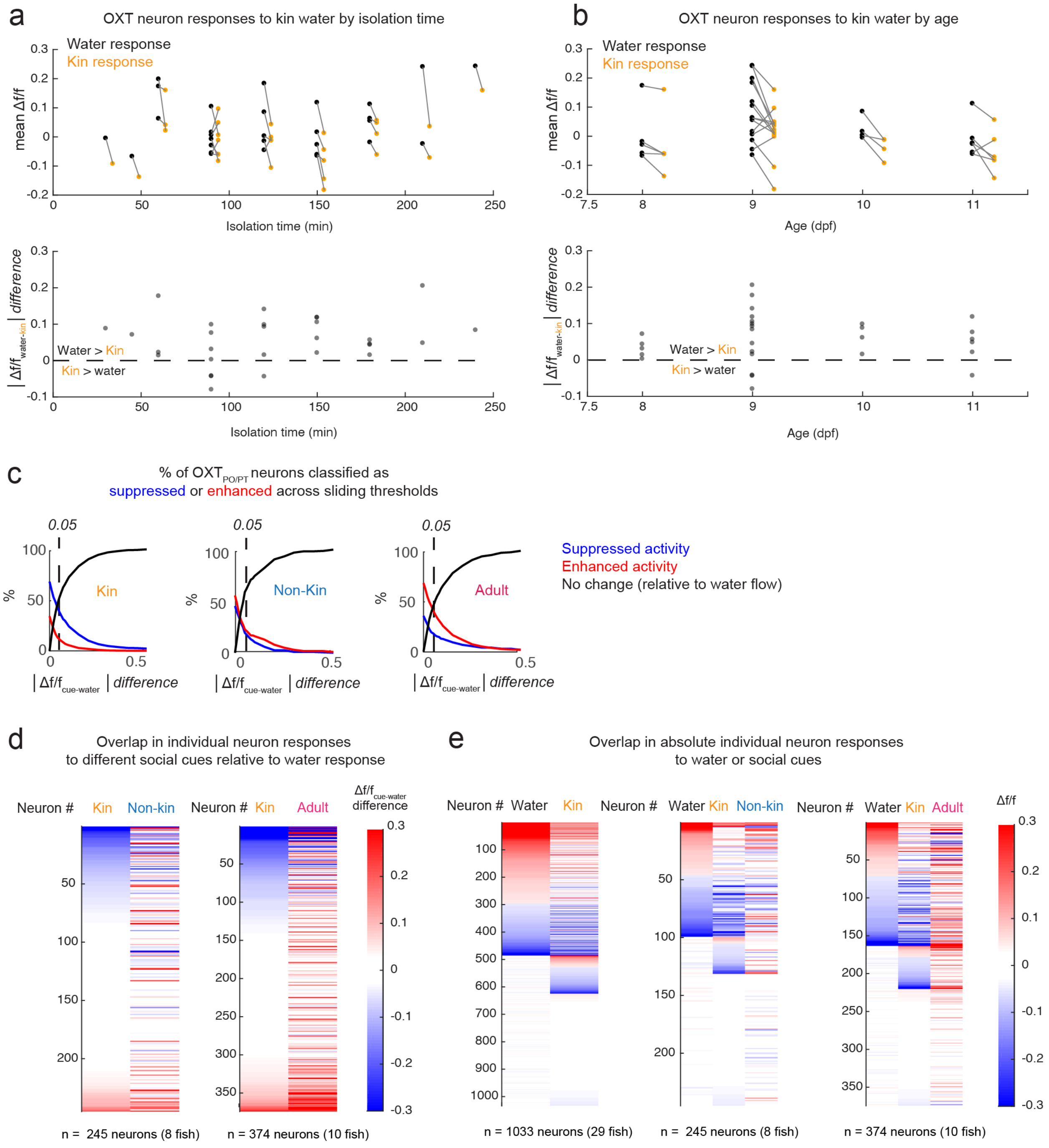

Supplementary Figure 2: Additional characterization of OXT neuron responses to social cues and water flow

a) Effect of kin water on OXT neuron activity plotted as a function of isolation time (min). Top panel: Average post-stimulus calcium activity (Δf/f) per fish (n = 29 fish) in response to either water flow (black) or kin water (orange). Bottom panel: Difference in water and kin water-induced Δf/f per fish. Fish were isolated for between 30 min to 4 hrs before imaging, though

the bulk of experiments were performed with 1.5 - 2.5 hrs isolation (note that time of isolation = time of embedding in agarose).

b) Effect of kin water on OXT neuron activity plotted as a function of age (dpf). Top panel: Average post-stimulus Δf/f per fish (n = 29 fish) in response to either water flow (black) or kin water (orange). Bottom panel: Difference in water and kin water-induced Δf/f per fish.

c) Percentage of OXT neurons that would be classified as showing suppressed (blue) or enhanced (red) activity by each water-borne cue, as a function of the mean difference in calcium activity from the water response (i.e., difference threshold). First, we quantified each neuron's activity as the mean Δf/f over a 60 second post-stimulus window. For each social cue, we then subtracted the same neuron's response to the control stimulus (i.e., water flow) to determine the mean difference relative to water flow. A difference threshold of 0.05 was used in subsequent panels.

d) OXT neurons sorted according to their differential response to kin water (relative to water flow) and their corresponding activities in response to non-kin or adult water. The data here is similar to Figure 2e, but not categorized into "suppressed" or "enhanced" classes.

e) Absolute calcium response (post minus pre-stimulus integrated Δf/f) of OXT neurons to water flow and other social cues, sorted according to their response to water flow.

Source data are provided as a Source Data file.

SUPPLEMENTARY FIGURE 3

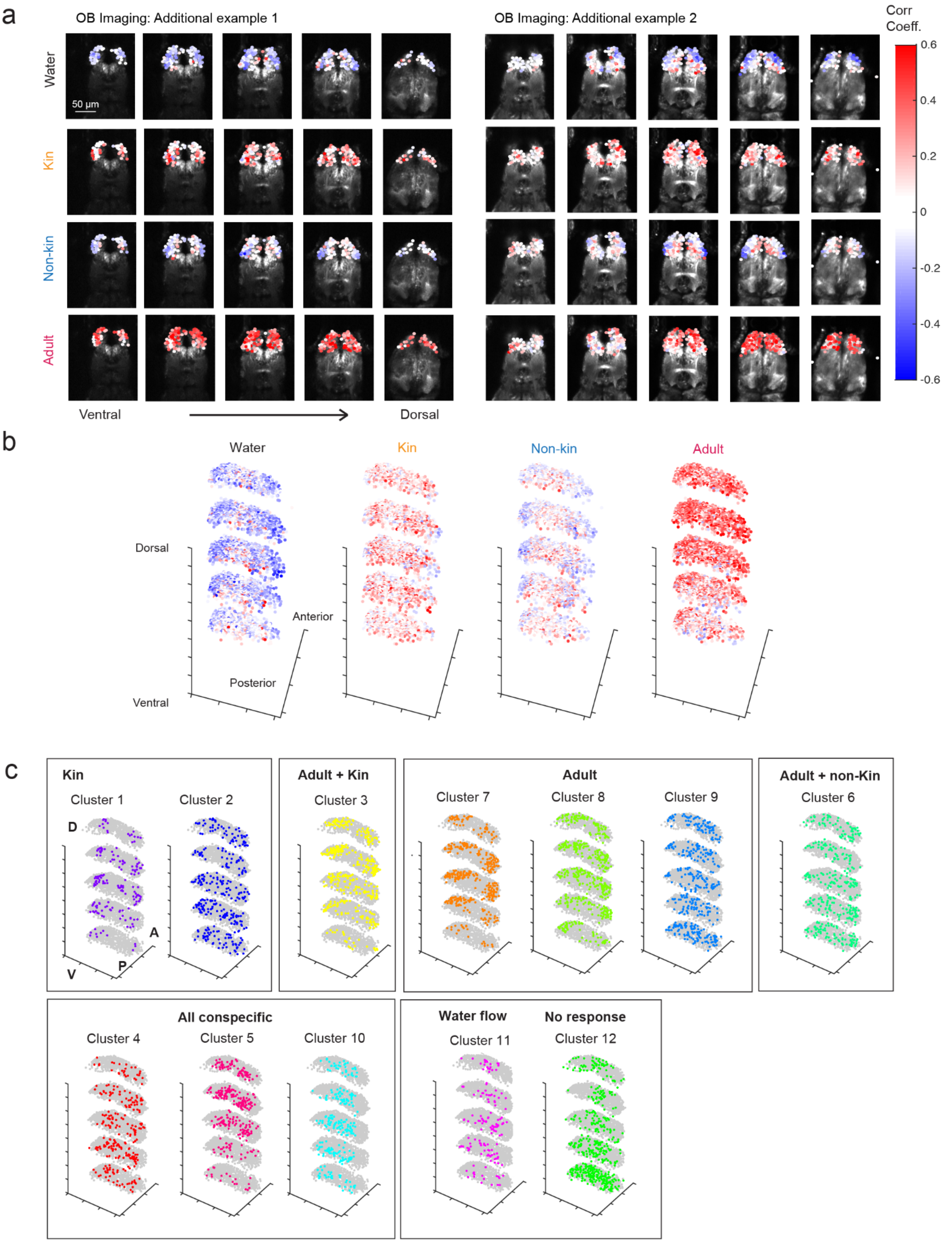

Supplementary Figure 3: The zebrafish olfactory bulb discriminates conspecific cues

a) Units extracted from two additional fish color coded (blue to red) by their coefficient correlations to each stimulus, and overlaid over anatomy images, per z-plane. Scale bar = 50 μm. This experiment was repeated on 10 fish with similar results.

b) 3D plot displaying correlation coefficients of all units across all 10 fish shown in Figure 3 to each stimulus. Color code same as in (a). As in Figure 3, x-y coordinates for all units per fish was scaled linearly to their minimum and maximum values in each dimension.

c) 3D plot displaying the spatial localization of units within each cluster. A = Anterior, P = Posterior, D = Dorsal, V = Ventral.

Source data are provided as a Source Data file.

SUPPLEMENTARY FIGURE 4

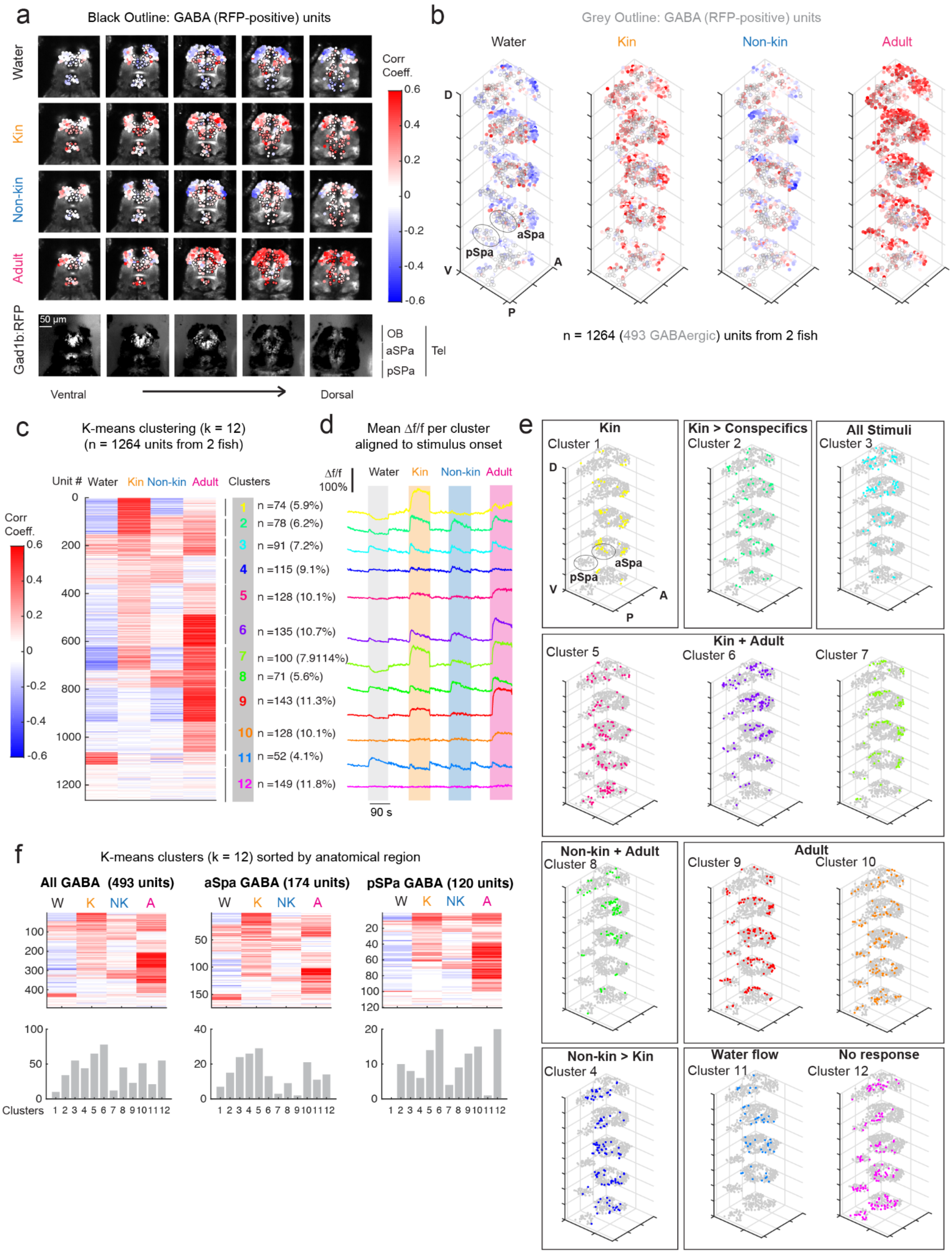

Supplementary Figure 4: GABAergic subpallial neurons respond to conspecific stimuli

a) Calcium imaging was performed on the telencephalon (including both OB and SPa) of transgenic fish (n = 2) co-expressing *Tg(HuC:GCaMP6s)* and *Tg(Gad1b:RFP)* as they were exposed to water flow, kin, non-kin, or adult water. Automatedly segmented units were identified as GABAergic (black outline) based on thresholded red fluorescence intensities (see Methods). Correlation coefficients for each unit with each stimulus regressor have been color coded (blue to red) and overlaid over the anatomy image per z-plane. Bottom-most row shows an anatomy stack of GABAergic cells per z-plane. GABAergic cells are present in the olfactory bulb, as well as anterior and posterior SPa, which is part of the ventral telencephalon. Scale bar = 50 μm.

b) 3D plot displaying correlation coefficients of all units from all fish to each stimulus. Color code is the same as in (a). As in Figure 2, XY coordinates for all units per fish were scaled linearly to their minimum and maximum values in each dimension. GABAergic units are outlined in grey. A = Anterior, P = Posterior, D = Dorsal, V = Ventral.

c) Correlation coefficients of all neurons (n = 1264 units from 2 fish) to each stimulus, sorted according to K-means cluster (k = 12). Number of units within each cluster and percentage representation of total units are displayed on the right.

d) Mean stimulus-triggered activity (Δf/f) for each cluster, aligned to stimulus onset. Order of clusters and units are the same as in (c).

e) 3D plot displaying the spatial localization of units within each cluster. Color code same as in (d). A = Anterior, P = Posterior, D = Dorsal, V = Ventral.

f) After k-means clustering (as in (c)), units were segmented into putative anatomical regions based on GABAergic identity and/or anterior-posterior location (aSPa located anteriorly but posterior to the OB, and pSPa cluster is posterior to the anterior commissure). Bar graphs show the number of units within each anatomical region belonging to each cluster.

Source data are provided as a Source Data file.

SUPPLEMENTARY FIGURE 5

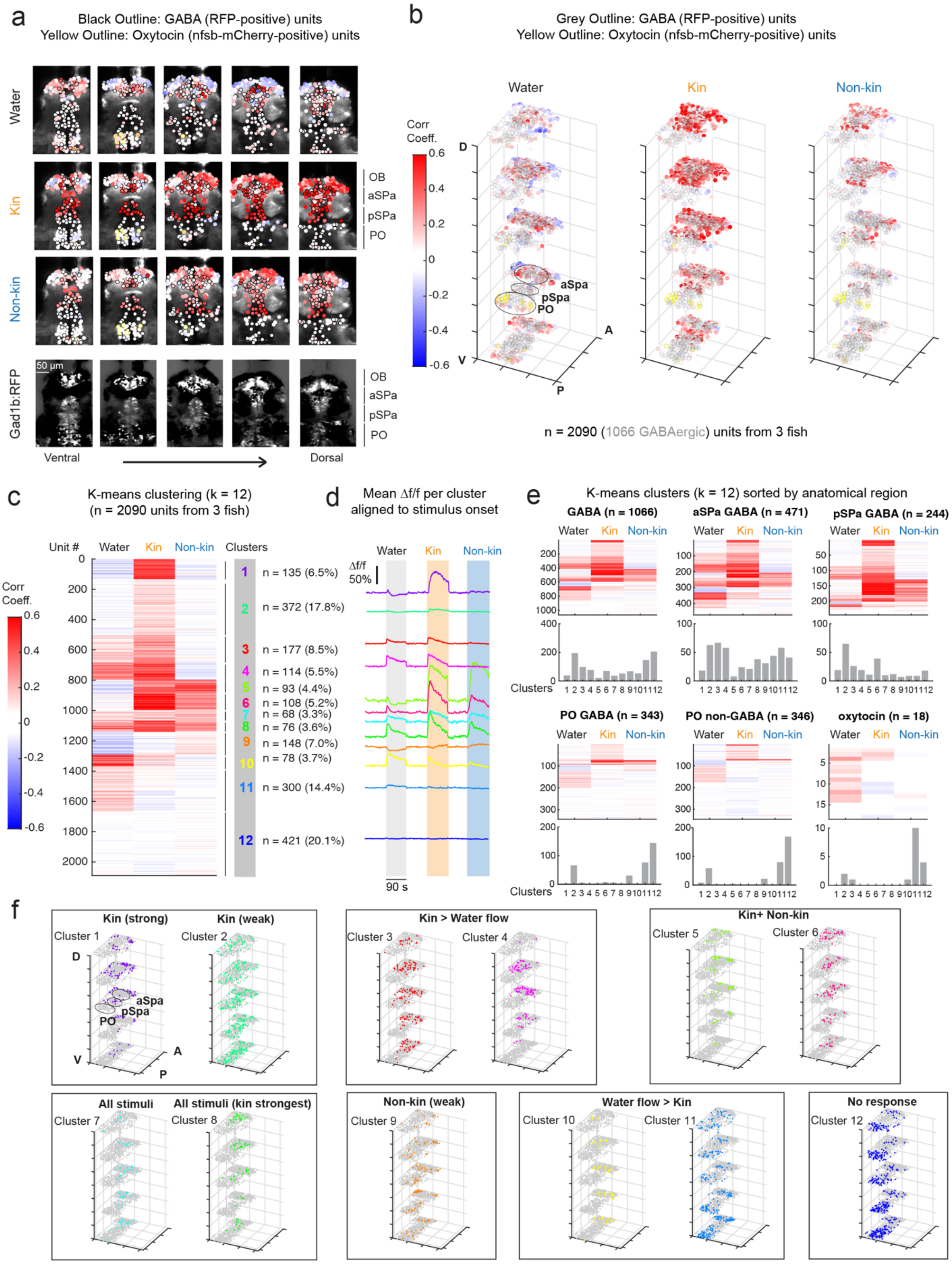

Supplementary Figure 5: GABAergic subpallial neurons discriminates kin and non-kin cues

a) Calcium imaging was performed on the telencephalons of transgenic fish (n = 3) co-expressing *Tg(HuC:GCaMP6s), Tg(Gad1b:RFP), and Tg(oxt:Gal4; UAS:nfsb-mCherry)*, as they were exposed to water flow, kin or non-kin water. Segmented units were identified as GABAergic (black outline) based on thresholded red fluorescence, whereas putative oxytocin neurons (yellow outline) were manually identified based on their brighter, more punctated labeling. Correlation coefficients for each unit with each stimulus regressor are color-coded (blue to red) and overlaid over the anatomy image per z-plane. Bottom-most row shows an anatomy stack of GABAergic cells per z-plane. GABAergic cells are present in the olfactory bulb, subpallium, and preoptic area. Scale bar = 50 µm.

b) 3D plot displaying correlation coefficients of all units across all fish to each stimulus. Color code same as in (a). XY coordinates for all units per fish were scaled linearly to their minimum and maximum values in each dimension. GABAergic units outlined in grey. A = Anterior, P = Posterior, D = Dorsal, V = Ventral.

c) Correlation coefficients of all neurons (n = 2090 units from 3 fish) to each stimulus, sorted according to K-means cluster (k = 12). Number of units within each cluster and percentage representation of total units are displayed on the right. Clusters specific to kin water and adult water, as well as clusters with mixed selectivity were observed. Clusters 1-4 show stronger responsiveness to kin cues over other cues. Note that clusters 5-8 are dominated by a single fish (shown in (a)) that had slightly stronger non-kin responses, the other 2 fish had weak non-kin responses (e.g. cluster 9).

d) Mean stimulus-triggered activity (Δf/f) for each cluster, aligned to stimulus onset. Order of clusters and units are the same as in (c).

e) After k-means clustering (as in (c)), units were segmented into putative anatomical regions based on GABAergic identity and/or anterior-posterior location (aSPa located anteriorly but posterior to the OB, and pSPa cluster is posterior to the anterior commissure). Bar graphs show the number of units within each anatomical region belonging to each cluster. Note that the PO region (including OXT neurons), unlike the SPa, predominantly has a stronger water flow response relative to kin or non-kin cues.

f) 3D plot displaying the spatial localization of units within each cluster. Color code same as in (e). A = Anterior, P = Posterior, D = Dorsal, V = Ventral.

Source data are provided as a Source Data file.

SUPPLEMENTARY FIGURE 6

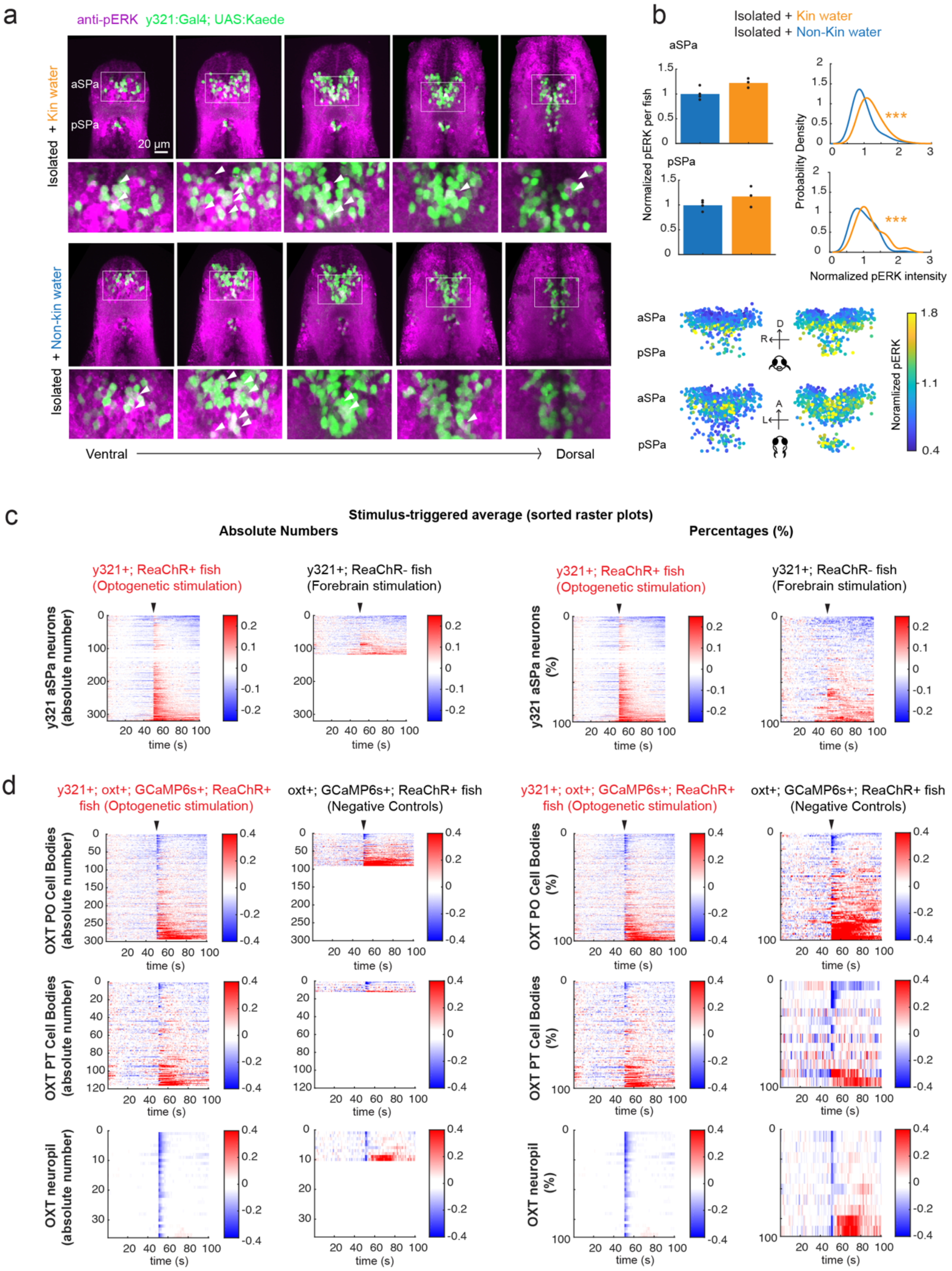

Supplementary Figure 6: y321 subpallial neurons are responsive to conspecific cues and sufficient to suppress OXT neuron activity

a) A subset of subpallial y321 cells overlap with anti-pERK staining following exposure to kin (top) or non-kin (bottom) water. Insets show the aSPa y321 neurons at higher magnification. Scale bar = 20 µm. This experiment was repeated on 3 and 4 fish for kin and non-kin water respectively with similar results.

b) Top: Quantification of pERK fluorescence in aSPa and pSPa y321 neurons after exposure to kin and non-kin water. When comparing the mean pERK signal per fish, there was no significant difference between kin (n= 3 fish) and non-kin (n = 4 fish) responses (aSPa: p = 0.11; pSPa: p = 0.40, two-sided Wilcoxon Rank-Sum Test). However, there was a significant right shift in pERK intensities in response to kin versus non-kin water (aSPa: ***p = $6.2x10^{-42}$, n 977 neurons (non-kin) / 637 neurons (kin)); pSPa: ***p = $1.4x10^{-4}$, n = 100 neurons (non-kin), n = 72 neurons (kin), two-sided Wilcoxon Rank-Sum Test). Bottom: Spatial distribution of y321 SPa neurons. Both the front view and top view are depicted (fish orientation shown in schematic), color coded by pERK intensity (yellow = highest intensity, blue = lowest intensity). 200 y321 neurons were randomly sampled per fish, with 600 neurons were drawn from this sample, to ensure even representation across all fish and groups. D = Dorsal, R = Right, A = Anterior, L = Left. Data are presented as mean values ± SEM.

c) Raster plots showing responses of individual y321 positive neurons to y321 optogenetic stimulation or a control stimulation (when ReaChR is not expressed). Laser pulse occurs at the 50 s mark (indicated by black arrow). Left panels show absolute cell number; right panels show percentage of cells.

d) Raster plots showing responses of individual OXT neurons in the PO or PT, or OXT neuropil regions, to y321 optogenetic stimulation or a control stimulation (when *Tg(y321:Gal4)* is not expressed). Laser pulse occurs at the 50 s mark (indicated by black arrow). Left panels show absolute cell number; right panels show percentage of cells.

Source data are provided as a Source Data file.

SUPPLEMENTARY FIGURE 7

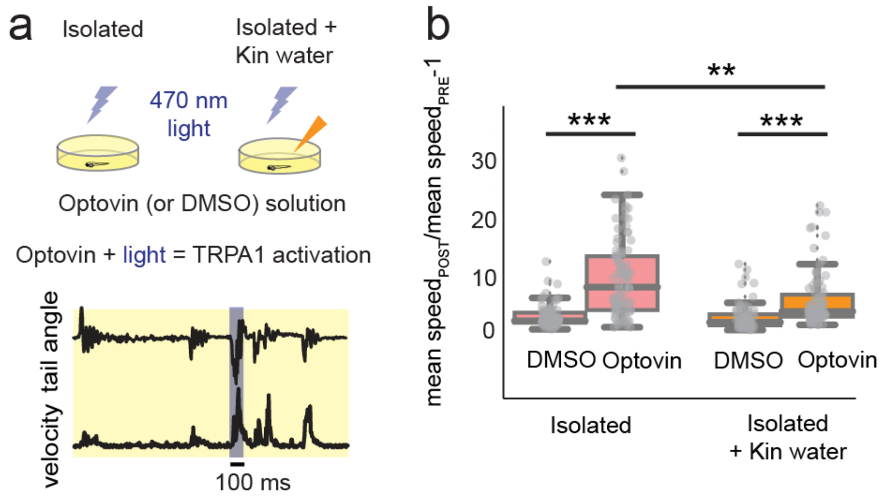

Supplementary Figure 7: Social context affects defensive behavior in larval zebrafish

a) Schematic for how the effect of kin-conditioned water on nocifensive behavior was tested. Fish were incubated with DMSO or Optovin solution, with or without kin-conditioned water. Short illumination with a 470 nm LED activates TRPA1 receptors and elicits large-angle tail bends and a corresponding increase in swim velocity.

b) The presence of conspecific cues significantly decreases TRPA1-induced velocity changes. DMSO (isolated) vs DMSO (kin water): p=0.55; Optovin (isolated) vs Optovin (kin water): **p = 0.0029; DMSO (isolated) vs Optovin (isolated): ***p = 1.1×10^{-8}, DMSO (kin water) vs Isolated (kin water): ***p = 8.0×10^{-5}; two-sided Wilcoxon Rank-Sum test (n = 42 (DMSO isolated) / 76 (Optovin isolated) / 43 (DMSO kin water) / 58 (Optovin kin water) bouts from n = 7 (isolated) / 7 (kin water) fish. Boxplot shows the median (center), 25th and 75th percentile or interquartile range (IQR, box), 1.5 IQRs of the lower and upper quartile (whiskers), and outliers beyond this range (diamonds), individual plots are overlaid.

Source data are provided as a Source Data file.

SUPPLEMENTARY FIGURE 8

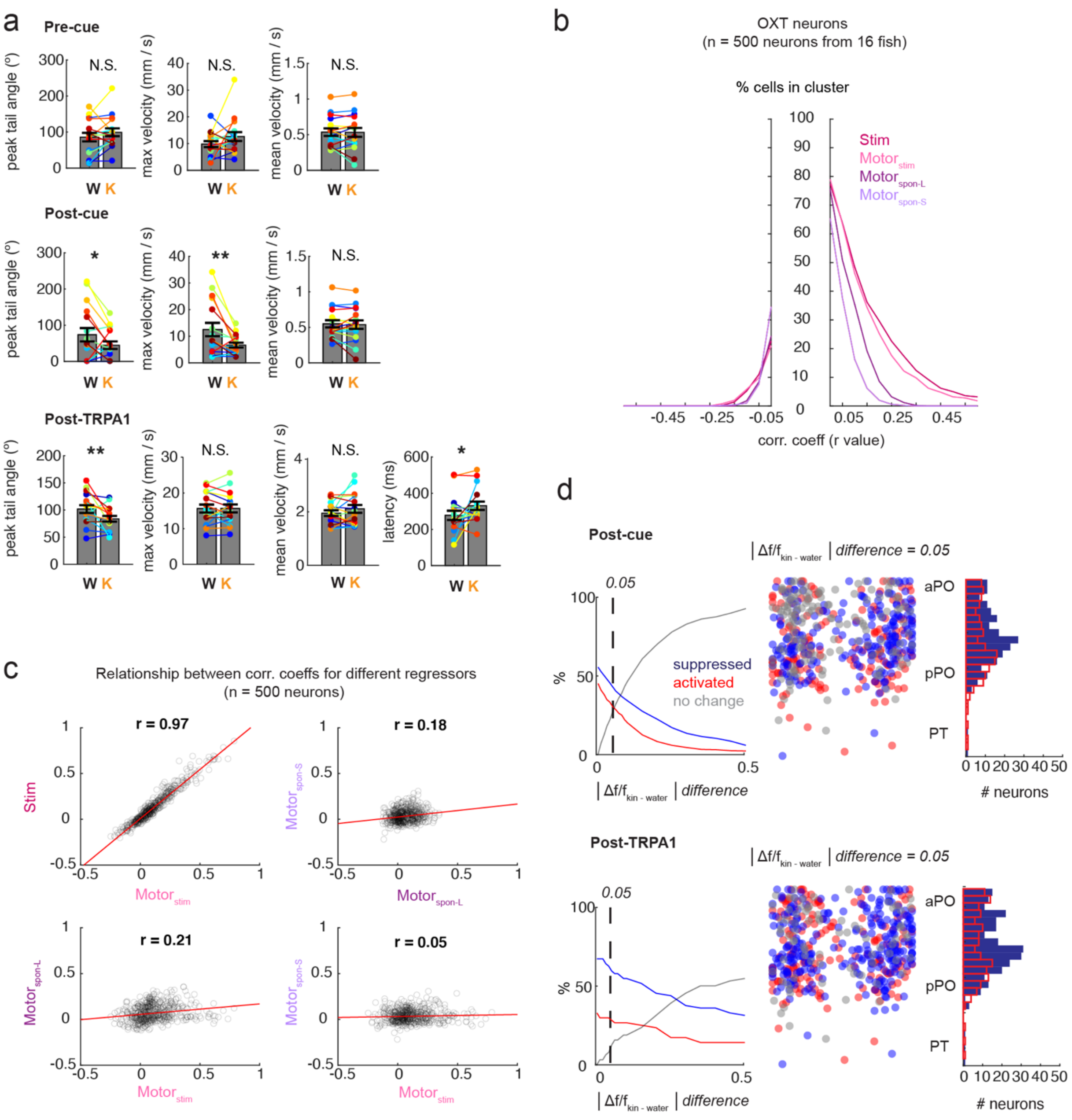

Supplementary Figure 8: Effect of kin water on behavior and OXT neuron activity

a) Effect of kin water on kinematics of swim bouts that occur either prior to cue delivery, post-cue delivery or directly after TRPA1 stimulation. Peak tail angle: $p = 0.95 / 0.038^* / 0.0061^{**}$; Maximum velocity: $p = 0.89 / 0.0075^{**} / 0.51$; Mean velocity: 0.67 / 0.51 / 0.80, response latency (post-TRPA1 stimulation): $p = 0.047^*$, one-sided Wilcoxon Signed-Rank test, n = 16 fish. Data are presented as mean values ± SEM. Each color represents an individual fish.

b) Percentage of cells which would be classified as being correlated with each regressor as a function of correlation coefficient, demonstrating strong OXT neuron correlation with TRPA1

stimulus and stimulus-induced behavior, and weaker correlation to spontaneous motor events.

c) Relationship between each OXT neuron’s correlation coefficients to different regressors.

d) Left: Percentage of OXT neurons that would be classified as suppressed (blue) or activated (red) by kin water, either post-cue (top) or post-TRPA1 (bottom) stimulation, as a function of the mean difference in integrated calcium activity from the water response (i.e., difference threshold). Center: spatial distribution of suppressed and activated neurons. Right: Distribution of suppressed and activated neurons along the A-P axis, using a threshold of 0.05.

Source data are provided as a Source Data file.
