# Supplementary material for: Social isolation modulates appetite and avoidance behavior via a common oxytocinergic circuit in larval zebrafish
Source: Nat Commun. 2022 May 11;13:2573. doi: 10.1038/s41467-022-29765-9 (PMC9095721; doi:10.1038/s41467-022-29765-9)
Supplement: Supplementary file 3 — Description of Additional Supplementary Information [file 41467_2022_29765_MOESM3_ESM.pdf]

Description of Additional Supplementary Information

Supplementary Movie 1: Z-stack (dorsal to ventral) of brain activity map shown in Figure 1a, overlaid on a *Tg(etVMAT:GFP)* brain used as an anatomical reference. Scale bar = 50 μm.

Supplementary Data 1: Z-brain anatomical regions that are more activated in isolated fish as compared to fish in a group. No regions were identified showing the opposite pattern (i.e., less active in isolated fish).
