# Supplementary material for: Social isolation modulates appetite and avoidance behavior via a common oxytocinergic circuit in larval zebrafish
Source: Nat Commun. 2022 May 11;13:2573. doi: 10.1038/s41467-022-29765-9 (PMC9095721; doi:10.1038/s41467-022-29765-9)
Supplement: Supplementary file 6 — Reporting Summary [file 41467_2022_29765_MOESM6_ESM.pdf]

nature portfolio

Corresponding author(s): Florian Engert

Last updated by author(s): 23 Jan, 2022

Reporting Summary

Nature Portfolio wishes to improve the reproducibility of the work that we publish. This form provides structure for consistency and transparency in reporting. For further information on Nature Portfolio policies, see our Editorial Policies and the Editorial Policy Checklist.

Statistics

For all statistical analyses, confirm that the following items are present in the figure legend, table legend, main text, or Methods section.

n/a	Confirmed	
☐	☒	The exact sample size (n) for each experimental group/condition, given as a discrete number and unit of measurement
☐	☒	A statement on whether measurements were taken from distinct samples or whether the same sample was measured repeatedly
☐	☒	The statistical test(s) used AND whether they are one- or two-sided<br>*Only common tests should be described solely by name; describe more complex techniques in the Methods section.*
☒	☐	A description of all covariates tested
☐	☒	A description of any assumptions or corrections, such as tests of normality and adjustment for multiple comparisons
☐	☒	A full description of the statistical parameters including central tendency (e.g. means) or other basic estimates (e.g. regression coefficient) AND variation (e.g. standard deviation) or associated estimates of uncertainty (e.g. confidence intervals)
☐	☒	For null hypothesis testing, the test statistic (e.g. F, t, r) with confidence intervals, effect sizes, degrees of freedom and P value noted<br>*Give P values as exact values whenever suitable.*
☒	☐	For Bayesian analysis, information on the choice of priors and Markov chain Monte Carlo settings
☒	☐	For hierarchical and complex designs, identification of the appropriate level for tests and full reporting of outcomes
☐	☒	Estimates of effect sizes (e.g. Cohen's d, Pearson's r), indicating how they were calculated

Our web collection on statistics for biologists contains articles on many of the points above.

Software and code

Policy information about availability of computer code

Data collection	Calcium imaging and tethered behavioral experiments were performed using custom LabView (2016 version 16.0) Software. Free-swimming behavioral data for Optovin stimulation was collected with custom Python (2.7) software.
Data analysis	Analysis of calcium imaging and behavior results was performed using MATLAB (R2017a) and Python (2.7 and 3.6). Additionally, feeding behavior and pERK were also analyzed using Fiji /Image J (2.0.0-rc-59/1.51n). Other external software utilized includes TurboReg (July 7, 2011 distribution), Suite2p (v0.7.1) and Raincloud plots (v1)

For manuscripts utilizing custom algorithms or software that are central to the research but not yet described in published literature, software must be made available to editors and reviewers. We strongly encourage code deposition in a community repository (e.g. GitHub). See the Nature Portfolio guidelines for submitting code & software for further information.

Data

Policy information about availability of data

All manuscripts must include a data availability statement. This statement should provide the following information, where applicable:

- Accession codes, unique identifiers, or web links for publicly available datasets
- A description of any restrictions on data availability
- For clinical datasets or third party data, please ensure that the statement adheres to our policy

Source data is provided with the manuscript as a Source Data File

Field-specific reporting

Please select the one below that is the best fit for your research. If you are not sure, read the appropriate sections before making your selection.

☒ Life sciences ☐ Behavioural & social sciences ☐ Ecological, evolutionary & environmental sciences

For a reference copy of the document with all sections, see nature.com/documents/nr-reporting-summary-flat.pdf

Life sciences study design

All studies must disclose on these points even when the disclosure is negative.

Sample size	No statistical methods were used to predetermine sample sizes but our sample sizes are similar to those reported in previous publications.
Data exclusions	No data was excluded from the analysis with the exception of data of poor quality (low signal to noise, motion artifacts) that would preclude analysis.
Replication	For calcium imaging experiments, a maximum of 4 to 5 fish could be imaged in a day, hence data was collected over multiple days / weeks, in more than 3 sessions, by 2 different experimenters. Results were reproducible across these conditions. Other experiments (e.g. pERK, feeding assays) were usually conducted by at least two experimenters over at least three sessions. For experiments conducted by only one experimenter, experiments were still conducted and reproducible over at least three sessions.
Randomization	In experiments that required randomization (e.g. pERK, feeding assay), larvae were randomly assigned to each condition. For experiments involving within-sample sample controls, stimulus delivery was randomized and delivered in an automated fashion.
Blinding	Data collection and analysis was not blinded as our processing pipeline is automated (no manual quantification involved).

Reporting for specific materials, systems and methods

We require information from authors about some types of materials, experimental systems and methods used in many studies. Here, indicate whether each material, system or method listed is relevant to your study. If you are not sure if a list item applies to your research, read the appropriate section before selecting a response.

Materials & experimental systems

n/a	Involved in the study	
☐	☒	Antibodies
☒	☐	Eukaryotic cell lines
☒	☐	Palaeontology and archaeology
☐	☒	Animals and other organisms
☒	☐	Human research participants
☒	☐	Clinical data
☒	☐	Dual use research of concern

Methods

n/a	Involved in the study	
☒	☐	ChIP-seq
☒	☐	Flow cytometry
☒	☐	MRI-based neuroimaging

Antibodies

Antibodies used	1) Rabbit anti-pERK and mouse anti-ERK (tERK) antibodies (Cell Signaling, #4370 and #4696, 1:500) 2) 647 goat anti-rabbit Alexafluor (Thermofisher A-21245) or 546 goat anti-rabbit Alexafluor (Thermofisher A-11035) or 647 goat anti-mouse Alexafluor (A-21235) or 546 goat anti-mouse Alexafluor (Thermofisher A-11030) (all at 1:500).
Validation	The anti-pERK and ERK antibodies were validated in Randlett et al, 2015.

Animals and other organisms

Policy information about studies involving animals; ARRIVE guidelines recommended for reporting animal research

Laboratory animals	5-8 dpf AB, Nacre (mitfa-/-) fish (in AB background) or WIK laboratory strains of zebrafish were used. Other transgenic lines used were generated on the AB background and are specified in the methods section.
Wild animals	The study did not involve wild animals.
Field-collected samples	The study did not involved field-collected samples.
Ethics oversight	IACUC at Harvard

Note that full information on the approval of the study protocol must also be provided in the manuscript.
